# Curcumin Protects against Ischemic Stroke by Titrating Microglia/Macrophage Polarization

**DOI:** 10.3389/fnagi.2017.00233

**Published:** 2017-07-21

**Authors:** Zongjian Liu, Yuanyuan Ran, Shuo Huang, Shaohong Wen, Wenxiu Zhang, Xiangrong Liu, Zhili Ji, Xiaokun Geng, Xunming Ji, Huishan Du, Rehana K. Leak, Xiaoming Hu

**Affiliations:** ^1^China-America Institute of Neuroscience, Beijing Luhe Hospital, Capital Medical University Beijing, China; ^2^Central Laboratory, Beijing Luhe Hospital, Capital Medical University Beijing, China; ^3^Institute of Hypoxia Medicine, Xuanwu Hospital, Xuan Wu Hospital of the Capital Medical University Beijing, China; ^4^Division of Pharmaceutical Sciences, Duquesne University Pittsburgh, PA, United States; ^5^Pittsburgh Institute of Brain Disorders and Recovery, and Department of Neurology, University of Pittsburgh School of Medicine Pittsburgh, PA, United States

**Keywords:** curcumin, microglial polarization, ischemic stroke, inflammation, neuroprotection

## Abstract

Stroke is the most common type of cerebrovascular disease and is a leading cause of disability and death. Ischemic stroke accounts for approximately 80% of all strokes. The remaining 20% of strokes are hemorrhagic in nature. To date, therapeutic options for acute ischemic stroke are very limited. Recent research suggests that shifting microglial phenotype from the pro-inflammatory M1 state toward the anti-inflammatory and tissue-reparative M2 phenotype may be an effective therapeutic strategy for ischemic stroke. The dietary phytochemical curcumin has shown promise in experimental stroke models, but its effects on microglial polarization and long-term recovery after stroke are unknown. Here we address these gaps by subjecting mice to distal middle cerebral artery occlusion (dMCAO) and administering curcumin intraperitoneally (150 mg/kg) immediately after ischemia and 24 h later. Histological studies revealed that curcumin post-treatment significantly reduced cerebral ischemic damage 3 days after dMCAO. Sensorimotor functions—as measured by the adhesive removal test and modified Garcia scores—were superior in curcumin-treated mice at 3, 5, 7 and 10 days after stroke. RT-PCR measurements revealed an elevation of M2 microglia/macrophage phenotypic markers and a reduction in M1 markers in curcumin-treated brains 3 days after dMCAO. Immunofluorescent staining further showed that curcumin treatment significantly increased the number of CD206^+^Iba1^+^ M2 microglia/macrophages and reduced the number of CD16^+^Iba1^+^ M1 cells 10 days after stroke. *In vitro* studies using the BV2 microglial cell line confirmed that curcumin inhibited lipopolysaccharide (LPS) and interferon-γ (IFN-γ)-induced M1 polarization. Curcumin treatment concentration-dependently reduced the expression of pro-inflammatory cytokines, including TNF-α, IL-6 and IL-12p70, in the absence of any toxic effect on microglial cell survival. In conclusion, we demonstrate that curcumin has a profound regulatory effect on microglial responses, promoting M2 microglial polarization and inhibiting microglia-mediated pro-inflammatory responses. Curcumin post-treatment reduces ischemic stroke-induced brain damage and improves functional outcomes, providing new evidence that curcumin might be a promising therapeutic strategy for stroke.

## Introduction

Stroke remains one of the leading causes of death and disability worldwide (Huuskonen et al., 2017; Mijajlović et al., 2017). Ischemic stroke accounts for approximately 80% of all strokes. The remaining 20% of strokes are hemorrhagic in nature. Thrombolytic therapy with recombinant tissue plasminogen activator (rtPA) is the only FDA-approved clinical treatment for acute ischemic stroke. A large number of neuroprotective agents have been investigated in the past few decades and shown promising results in animal models of stroke; all of them, however, failed in subsequent clinical trials (Cook et al., 2012; Guekht et al., 2017). Thus, rescuing neurons without improving the microenvironment in the injured brain is not sufficient to achieve long-term protection and functional recovery after stroke. Therefore, new therapeutic strategies that re-establish brain homeostasis and foster a permissive environment for cell survival or regeneration are being actively explored.

Microglia and infiltrated macrophages play critical roles in regulating immune and inflammatory responses after brain injuries (Perry et al., 2010). Accumulating evidence shows that microglia/macrophages assume different phenotypes with distinct functions during the course of ischemic brain injury (Hu et al., 2012). For example, alternatively activated M2 microglia protect neighboring cells by removing cell debris and releasing trophic factors for brain repair. However, classically activated M1 microglia may exacerbate brain injury by producing neurotoxic substances when overactivated for prolonged times, even if they participate in clearing cell debris at early stages after stroke (Hu et al., 2012, 2015). These two microglia/macrophage phenotypes probably lie along a continuum of activation status. Such phenotypic plasticity and diversity support the view that microglia serve a unique and important role in maintaining brain homeostasis under physiological and pathological conditions. Several agents known to be protective against ischemic stroke, such as Ginkgolide B (Shu et al., 2016), Malibatol A (Pan et al., 2015), thiamet G (He et al., 2016), and Exendin-4 (Darsalia et al., 2014) have the capacity to promote M2 polarization in microglia. Thus, balancing microglia/macrophage phenotype is a promising therapeutic strategy for stroke treatment.

Curcumin (1,7-bis[4-hydroxy-3-methoxyphenyl]-1,6-heptadiene-3,5-dione) is a major component of the rhizomes of *Curcuma longa* and a well-established polyphenolic antioxidant (Gupta et al., 2012). A number of studies have demonstrated that curcumin can protect against ischemic stroke in experimental models (Thiyagarajan and Sharma, 2004; Lapchak, 2011; Shah et al., 2016). Pre-/post-stroke treatment with curcumin was found to effectively reduce infarct volumes and improve functional outcomes. Curcumin has also been proposed as a promising agent for stroke prevention in humans (Ovbiagele, 2008). Manifold mechanisms are involved in the protective effects of curcumin, including anti-oxidative, anti-inflammatory (Thiyagarajan and Sharma, 2004; Yang et al., 2009; Wu et al., 2013), and anti-apoptotic mechanisms (Zhao et al., 2008, 2010; Altinay et al., 2017), as well as neurogenesis (Liu S. et al., 2016). However, the effect of curcumin on microglial phenotypic polarization after ischemic stroke has not been explored.

In the present study, we assessed the effects of curcumin on microglial polarization and inflammatory responses both *in vitro* and in a mouse model of ischemic stroke. Our results demonstrate that curcumin promoted microglial M2 polarization and inhibited M1 polarization, both *in vivo* and *in vitro*. Furthermore, curcumin treatment ameliorated post-stroke brain injury and improved functional outcomes. These findings support the view that curcumin improves functional recovery after stroke by adjusting the balance between M1 and M2 microglial states.

## Materials and Methods

### Animals

Adult male C57BL/6 mice (8–10 weeks, 23–25 g) were purchased from the Vital River Laboratory Animal Technology Co., Ltd (Beijing, China). All animal experiments were approved by the Institutional Animal Care and Use Committee of Capital Medical University. Food and water were available *ad libitum*. All efforts were made to minimize animal suffering and the number of animals used. Animals were randomly divided into: (1) sham; (2) stroke plus vehicle; and (3) stroke plus curcumin groups. In this study, a total of 80 mice were used for infarct volume measurements (23 mice), behavioral tests and immunohistochemical examinations (42 mice), and mRNA or protein expression measurements (15 mice).

### Distal Middle Cerebral Artery Occlusion (dMCAO) Model

Anesthesia was induced with 2% isoflurane in 70% nitrogen/30% oxygen gas mixture. Rectal temperature was maintained at 37 ± 0.5°C with a heating pad. A ~2 cm incision was made between the right eye and ear. Using a surgical microscope, the temporal muscle was dissected to expose right zygomatic arches and squamosal bone. A craniotomy was performed and the right MCA was occluded distal to the lenticulostriate branches with bipolar electrocautery (Goldbov Photoelectronics CO. Ltd, Wuhan, China). The middle cerebral artery occlusion (MCAO) was accompanied by 15-min bilateral occlusion of the common carotid artery (CCA). Regional cerebral blood flow (rCBF) was monitored with a two-dimensional laser speckle imager (Perimed AB, Järfälla, Sweden). Mice with rCBF reduction less than 30% of baseline levels were excluded from further experiments. Sham-operated mice were manipulated in the same way, but the MCA and CCA were not occluded.

### Drug Preparation and Treatments

Curcumin (Sigma-Aldrich) was dissolved in 5 mol/L NaOH, titrated to pH 7.4 using 1 mol/L HCl, and then diluted with saline. Ischemic mice were subjected to intraperitoneal injections of 150 mg/kg curcumin or the same volume of vehicle. Injections were performed 0 h and 24 h after reperfusion of the CCA.

### Measurements of Infarct Volume

At 72 h after cerebral ischemia, mice were decapitated and brains were rapidly removed on ice. Brains were sliced into 1 mm-thick coronal sections and stained with 2% 2, 3, 5-triphenyltetrazolium chloride (TTC, Sigma-Aldrich), as previously described (Liu X. et al., 2016). The infarct area was calculated as the area of the contralateral hemisphere minus the noninfarcted area of the ipsilateral hemisphere by a person blinded to the experiment group. Infarct volumes were determined using National institutes of Health ImageJ software. The total infarct areas were multiplied by the thickness of the brain sections to obtain the infarct volumes.

### Adhesive Removal Test

Sensorimotor functional recovery after stroke was measured before or 3, 5, 7 and 10 days after stroke with the adhesive removal test, as described in prior studies (Wang et al., 2017). In brief, a mouse was placed in a cage for 1 min. An adhesive tape (50 mm^2^) was applied to the distal radial region of the right forelimb as a tactile stimulus. The time to contact and the time to remove the tape were both recorded. Each animal was tested three times with a cutoff time of 120 s per trial. The data are presented as the mean time to contact and the mean time to remove the tape on each testing day. The investigators performing the assessments were blinded to experimental group assignments.

### Modified Garcia Score Test

The Modified Garcia Score system was used to assess sensorimotor functions at 3, 5, 7 and 10 days after dMCAO by an observer blinded to the experimental groups. Five tests were performed: body proprioception, vibrissae touch, limb symmetry, lateral turning and forelimb walking with scores of 0–3 for each test, as previously reported studies (Wang et al., 2017). The data from pre-stroke tests were defined as the baseline.

### Immunohistochemistry and Cell Counting

Brain slices were prepared and subjected to immunohistochemistry, as previously published (Pan et al., 2015). Mice were perfused with saline and 4% paraformaldehyde. Brains were then removed, followed by cryoprotection in 30% sucrose. The brains were sliced into 20 μm-thick coronal sections at −25°C using a freezing microtome (CM3050S, Leica, German). The slices were then subjected to immunofluorescent staining. Non-specific staining was blocked with 10% normal serum. Next, brain slices were incubated with mouse anti-CD16 (1:100, BD Pharmingen, San Jose, CA, USA), goat anti-CD206 (1:100, R&D Systems, Minneapolis, MN, USA), and rabbit anti-Iba-1 (1:100, Wako, Richmond, VA, USA) overnight at 4°C. The sections were then treated with fluorophore-conjugated secondary antibodies (Invitrogen Corporation, Carlsbad, CA, USA)—goat anti-rabbit antibody conjugated to Alexa 594 (1:400), goat anti-mouse antibody conjugated to Alexa 488 (1:400), and donkey anti-goat antibody conjugated to Alexa 488 (1:400), at room temperature for 2 h. All images were captured with a fluorescence microscope (DM4000 B LED, Leica Microsystem, Germany) and analyzed by a blinded observer with ImageJ. Cell numbers were calculated from three randomly-selected microscopic fields, and three consecutive sections were analyzed for each brain.

### BV2 Microglia Cell Line Cultures

The BV2 microglia cell line (Cell Center, Institute of Basic Medical Sciences, CAMS and PUMC, Beijing, China) was cultured in RPMI 1640 (Cat No.: C11875500BT, Gibco), supplemented with 10% fetal bovine serum (Cat No.: 16000-044, Gibco) and 1% penicillin-streptomycin (Cat No.: 15070-063, Gibco). Cells were incubated at 37°C in a humidified atmosphere containing 5% CO_2_ and the medium was changed every 2 days. For M1 stimulation, lipopolysaccharide (LPS, 100 ng/mL) and IFN-γ (20 ng/mL) were added to BV2 microglia cultures. Different concentrations of curcumin were added to the cultures immediately following the application of M1 inducers. After 48 h of treatment, cells were collected for RT-PCR and the supernatant was harvested for the detection of cytokines by ELISA.

The cytotoxicity of curcumin against BV2 microglia cell line was analyzed by a lactate dehydrogenase (LDH) cytotoxicity assay kit (Cat No.: C0017, Beyotime, Shanghai, China) according to the manufacturer’s protocol. Absorbance was measured at 490 nm by a microplate reader (Cat No.: Synergy HT, Biotek, Winooski, VT, USA). All cell culture experiments were performed in duplicate and repeated six times.

### Real-Time PCR

Total RNA was extracted from the cortex of C57BL/6 mice or from the BV2 microglia cell line (Cell Center, Institute of Basic Medical Sciences, CAMS and PUMC, Beijing, China) by RNeasy® Lipid Tissue Mini Kit (Cat No.: 74804, QIAGEN) according to manufacturer’s instructions. Three microgram RNA were reverse-transcribed into cDNA using the SuperScript^™^ III First-Strand Synthesis SuperMix for qRT-PCR (Cat No.: 11752-250, Invitrogen). RT-PCR was performed using quantitative PCR systems (Applied Biosystems® 7500 Real-Time PCR Systems, Thermo Fisher Scientific, Waltham, MA, USA) with corresponding primers (Table 1, Invitrogen) and a fluorescent dye (RT2 SYBR® Green FAST Mastermixes, Cat No.: 330603, QIAGEN). The cycle time (CT) was normalized to GAPDH in the same sample. The expression levels of mRNAs were reported as fold changes vs. sham control.

**Table 1:** Primers for RT-PCR.

Genes		Primers (5′-3′)
GAPDH	Forward	AGGTCGGTGTGAACGGATTTG
	Reverse	GGGGTCGTTGATGGCAACA
IL-12	Forward	AAATGAAGCTCTGCATCCTGC
	Reverse	TCACCCTGTTGATGGTCACG
TNF-α	Forward	GATCTCAAAGACAACCAACTAGTG
	Reverse	CTCCAGCTGGAAGACTCCTCCCAG
iNOS	Forward	CAAGCACCTTGGAAGAGGAG
	Reverse	AAGGCCAAACACAGCATACC
CD16	Forward	TTTGGACACCCAGATGTTTCAG
	Reverse	GTCTTCCTTGAGCACCTGGATC
CD32	Forward	AATCCTGCCGTTCCTACTGATC
	Reverse	GTGTCACCGTGTCTTCCTTGAG
CD206	Forward	CAAGGAAGGTTGGCATTTGT
	Reverse	CCTTTCAGTCCTTTGCAAGC
Arg1	Forward	TCACCTGAGCTTTGATGTCG
	Reverse	CTGAAAGGAGCCCTGTCTTG
Ym1/2	Forward	CAGGGTAATGAGTGGGTTGG
	Reverse	CACGGCACCTCCTAAATTGT

### ELISA Measurements

Cytokine production (IL-1β, IL-2, IL-12p70, IL-13 and TNF-α) in the BV2 culture media was determined by ELISA (Cusabio Co., Ltd., Wuhan, China), according to the manufacturer’s instructions. Each sample was assayed in triplicate.

### Statistical Analyses

Power analyses were used to determine the number of mice within each experimental group, according to our past experience with similar measurements (*α* = 0.05 and *β* = 0.20). All data are presented as mean ± SEM. The Student’s two tailed *t*-test was used for comparisons of two experimental groups. Differences in means across multiple groups were analyzed using one-way or two-way ANOVA, depending on the number of independent variables. Differences in means across multiple groups with multiple measurements over time were analyzed using two-way repeated measures ANOVA. When the ANOVA revealed significant differences, the Bonferroni *post hoc* test was used for pairwise comparisons between means.

## Results

### Curcumin Treatment Significantly Reduces Infarct Volumes 3 days after dMCAO

**Figure 1:**
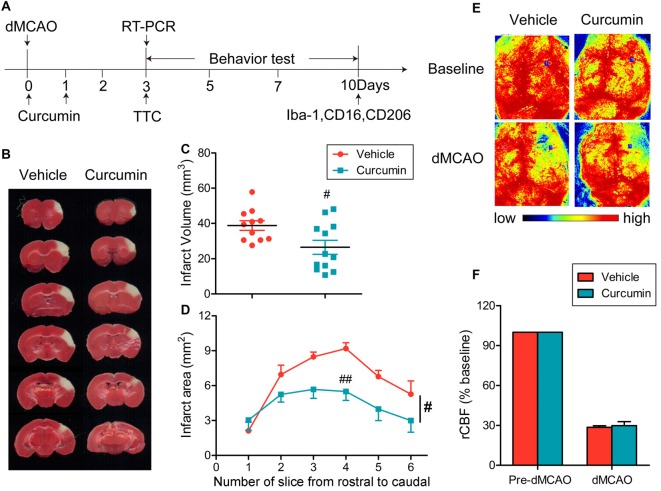
Curcumin treatment reduces infarct volume 3 days after distal middle cerebral artery occlusion (dMCAO). **(A)** Timeline for *in vivo* experiments. Stroke was induced in mice with permanent occlusion of the dMCAO. Curcumin was intraperitoneally injected (150 mg/kg) at 0 and 24 h after ischemia. At 3 days after stroke, RT-PCR was performed to determine the mRNA expression of M1/M2 microglia markers and infarct volume was measured after 2,3,5-triphenyltetrazolium chloride (TTC) staining. Adhesive removal tests and modified Garcia scores were used to measure post-stroke sensorimotor functions at 0, 3, 5, 7 and 10 days after cerebral ischemia. To confirm whether curcumin regulates microglial polarization in the brain after dMCAO, brain sections were double-stained for Iba-1 (microglia) with CD206 (M2 marker) or CD16 (M1 marker) at 10 days after stroke. **(B)** Representative TTC staining at 3 days after dMCAO. **(C)** Infarct volume in vehicle (red) and curcumin-treated (blue) dMCAO mice. **(D)** Infarct areas in TTC-stained slices. **(E)** Representative two-dimensional laser speckle images for regional cerebral blood flow (rCBF) in curcumin and vehicle-treated mice. **(F)** Changes in rCBF in infarct regions. Data are expressed as a percentage of pre-dMCAO rCBF. *n* = 11 animals per group. Data are means ± SEM. ^#^*p* < 0.05, ^##^*p* < 0.01 vs. vehicle-treated group, Student’s two-tailed *t* test **(C)** and two-way ANOVA followed by Bonferroni *post hoc* test **(D)**.

Curcumin (150 mg/kg) was administered intraperitoneally immediately after dMCAO and 24 h after surgery (Figure 1A). As shown in Figures 1B–D, curcumin treatment significantly reduced infarct volumes compared to vehicle. rCBF was measured before and after dMCAO with a two-dimensional laser speckle imager (Figure 1E). For all animals assigned to curcumin or vehicle groups, the rCBF after dMCAO was reduced to about 30% of pre-ischemic baseline values (Figure 1E). There was no statistical difference in rCBF reduction between curcumin and vehicle-treated groups (Figure 1F), verifying that outcome differences between the experimental and control groups cannot be attributed to different degrees of the original ischemic injury.

### Curcumin Treatment Significantly Improves Sensorimotor Functions after dMCAO

**Figure 2:**
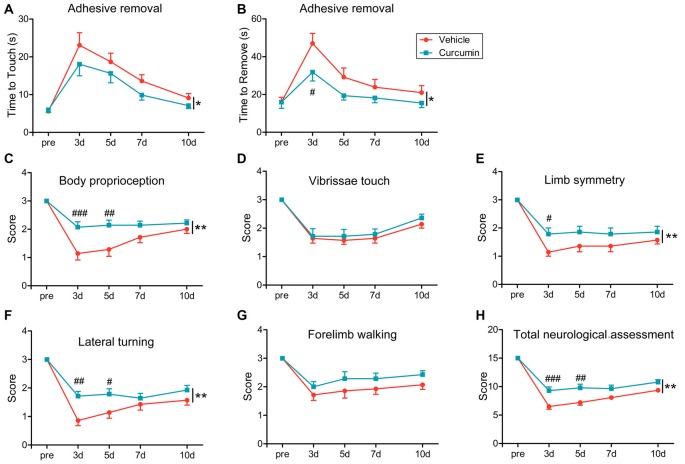
Curcumin treatment significantly improves sensorimotor functions early after dMCAO. Adhesive removal test **(A,B)** and modified Garcia scores **(C–H)** were used to evaluate sensorimotor functions pre-surgery or 3, 5, 7 and 10 days after dMCAO in mice treated with vehicle or curcumin. **(A)** Time to touch adhesive tape. **(B)** Time to remove adhesive tape. **(C)** Body proprioception. **(D)** Vibrissae touch. **(E)** Limb symmetry. **(F)** Lateral turning. **(G)** Forelimb walking. **(H)** Total neurologic assessment score. *n* = 14 animals per group. Data are means ± SEM. *p < 0.05, **p < 0.01 between vehicle-treated group and curcumin-treated group. ^#^*p* < 0.05, ^##^*p* < 0.01, ^###^*p* < 0.001 at a particular time point between vehicle-treated and curcumin-treated group. Two way repeated measures ANOVA followed by Bonferroni *post hoc* test.

To evaluate the effects of curcumin treatment on functional outcomes after dMCAO, sensorimotor deficits were measured by the adhesive removal test and modified Garcia score system at 3, 5, 7 and 10 days post-stroke. Curcumin treatment significantly improved neurological performance in the adhesive removal test after dMCAO, as manifested by a consistent reduction in the times to contact and then remove the tape from the compromised limb (Figures 2A,B, respectively). In addition, sensorimotor functions such as body proprioception (Figure 2C), limb symmetry (Figure 2E), and lateral turning (Figure 2F) were all significantly improved in curcumin-treated mice compared to vehicle-treated mice, resulting in an increase in the total neurological score, especially at day 3–5 after stroke (Figure 2H). There was no significant difference in vibrissae touch (Figure 2D) or forelimb walking (Figure 2G) between vehicle and curcumin-treated groups. These data reveal improvements in some aspects of neurological function at early timepoints after stroke with curcumin.

### Curcumin Treatment Inhibits M1 Polarization and Promotes M2 Polarization of Microglia

**Figure 3:**
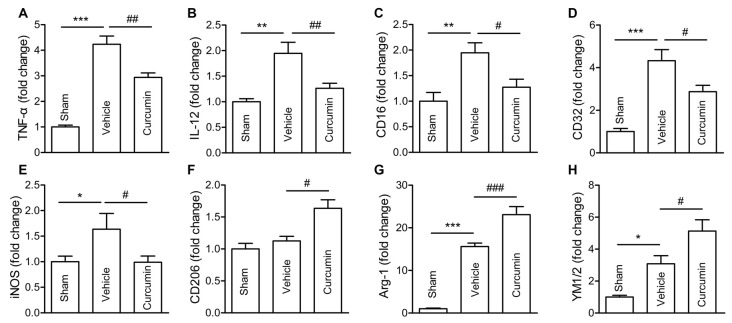
Curcumin treatment inhibits M1 polarization and promotes M2 polarization of microglia/macrophages 3 days after dMCAO. Mice were subjected to dMCAO. Curcumin (150 mg/kg) or the same volume of vehicle was intraperitoneally injected into mice at 0h and 24 h after dMCAO. Brain samples were collected 3 days after dMCAO. mRNA expression of M1 microglia/macrophage signature genes (TNF-α **(A)**, IL-12 **(B)**, CD16 **(C)**, CD32 **(D)**, and iNOS **(E)**) and M2 signature genes (CD206 **(F)**, Arg-1**(G)**, and YM1/2 **(H)**) were measured by RT-PCR. Data are means ± SEM. *n* = 5 animals per group. *p < 0.05, **p < 0.01, ***p < 0.001 vs. sham; ^#^*p* < 0.05, ^##^*p* < 0.01, ^###^*p* < 0.001 vs. vehicle-treated, one-way ANOVA followed by Bonferroni *post hoc* test.

Polarization of microglia plays a critical role in the pathological progression of ischemic stroke (Hu et al., 2012). Thus, the effect of curcumin on microglial polarization was examined by RT-PCR. RNA samples were prepared from dMCAO mice treated with curcumin or vehicle. As shown in Figure 3, the expression of M1 markers (TNF-α, IL-12, CD16, CD32 and iNOS) and M2 markers (Arg-1 and YM1/2) were all significantly increased in vehicle-treated stroke mice 3 days after dMCAO. Curcumin treatment significantly inhibited this increase of M1 markers and enhanced the expression of M2 markers 3 days after dMCAO (Figure 2).

**Figure 4:**
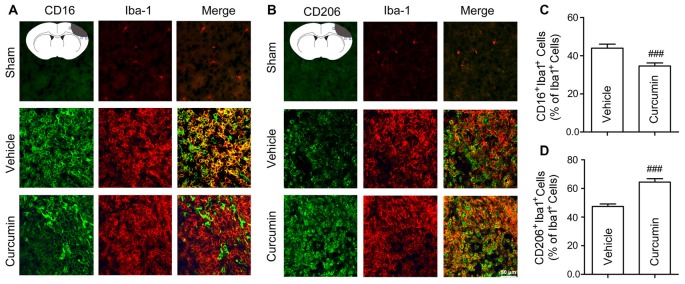
Curcumin treatment enhances M2 polarization and suppresses M1 polarization of microglia in the ischemic cortex at 10 days after dMCAO. Representative double-immunofluorescence staining for CD16 or CD206 and Iba-1 markers in brain sections obtained from curcumin or vehicle-treated mice 10 days after dMCAO, or from sham-operated mice. Scale bar: 50 μm. Blue squares in the schematic diagram illustrate the anatomical location of images in the ipsilateral peri-infarct cortex. **(A)** Cortex sections co-stained for CD16 (M1 marker) (green) and Iba-1(red). **(B)** Cortex sections co-stained for CD206 (M2 marker) (green) and Iba-1 (red). **(C)** Quantification of the percentage of CD16^+^Iba-1^+^ cells among total Iba-1^+^ cells. **(D)** Quantification of the percentage of CD206^+^/Iba-1^+^ cell among total Iba-1^+^ cells. Data are means ± SEM. *n* = 6 animals per group. ^###^*p* < 0.001 vs. vehicle-treated group, Student’s two-tailed *t* test.

To further assess whether curcumin regulates microglial polarization in the brain after dMCAO, brain sections were double-stained for Iba-1 (microglial marker) and CD16 (M1 marker) or CD206 (M2 marker). As shown in Figures 4A,C, the percentage of CD16^+^Iba-1^+^ cells among total Iba-1^+^ microglia/macrophages was significantly higher in vehicle-treated mice compared to curcumin-treated stroke mice. Moreover, curcumin administration significantly increased the percentage of CD206^+^Iba-1^+^ M2 microglia/macrophages after dMCAO (Figures 4B,D). Taken together, these results demonstrate that curcumin treatment promotes M2 polarization and inhibits M1 polarization after stroke, consistent with its neuroprotective properties.

### Administration of Curcumin Promotes M2 Polarization and Inhibits M1 Polarization *In Vitro*

**Figure 5:**
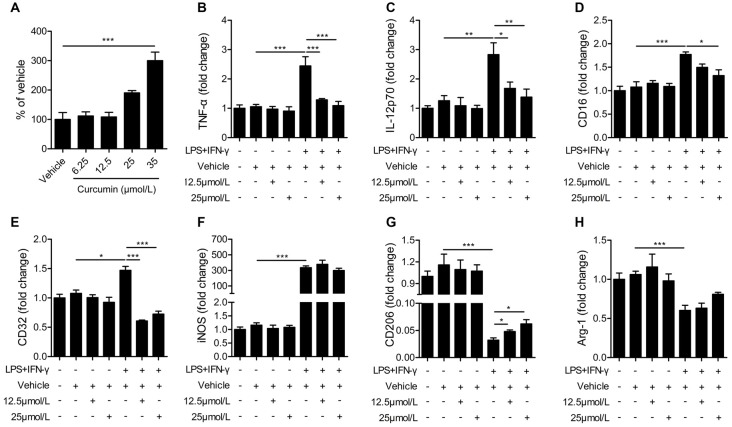
Curcumin inhibits M1 polarization and promotes M2 polarization in lipopolysaccharide (LPS) and interferon-γ (IFN-γ)-activated BV2 microglial cells. **(A)** lactate dehydrogenase (LDH) cell cytotoxicity assay in BV2 cells cultured with vehicle or 6.25, 12.5, 25 and 35 μmmol/L curcumin. **(B–H)** BV2 microglia were stimulated with LPS (100 ng/mL) and IFN-γ (20 ng/mL), and then treated with vehicle or 12.5 and 25 μmmol/L curcumin. The mRNA expression of M1 markers (TNF-α **(A)**, IL-12p70 **(B)**, CD16 **(C)**, CD32 **(D)** and iNOS **(E)**) and M2 markers (CD206 **(G)** and Arg-1 **(H)**) were examined by RT-PCR. Data are means ± SEM. *n* = 6 per group. Samples were collected from six independent experiments, each performed in duplicate. *p < 0.05, **p < 0.01, ***p < 0.001, one-way ANOVA followed by Bonferroni *post hoc* test.

The BV2 microglial cell line was used to further determine the effects of curcumin on microglial polarization. First, we performed the LDH assay to determine curcumin cytotoxicity in BV2 microglia cells (Figure 5A). Treatment with 6.25, 12.5 and 25 μmmol/L curcumin showed no significant effects on microglial survival. Treatment with 35 μmmol/L curcumin exerted a significantly cytotoxic effect on BV2 microglia cells. Therefore, the 12.5 and 25 μmol/L concentrations were selected for further experiments.

BV2 microglial cells were treated with LPS (100 ng/mL) and IFN-γ (20 ng/mL) for 48 h to induce the M1 phenotype. As shown in Figures 5B–E, the mRNA expression of M1 markers (TNF-α, IL-12, CD16 and CD32) was increased significantly in BV2 microglia after stimulation with LPS and IFN-γ, but markedly reduced after treatment with 12.5 or 25 μmmol/L curcumin. However, the mRNA expression of iNOS (M1 marker, Figure 5F) was drastically upregulated by LPS and IFN-γ and not significantly reduced with curcumin. In contrast to the upregulation of M1 markers with LPS and IFN-γ, the mRNA expression of M2 markers (CD206 and Arg-1) was reduced by these two pro-inflammatory stimuli. The 12.5 or 25 μmmol/L concentrations of curcumin partially reversed this reduction of CD206 expression (Figure 5G), but showed only marginal effects on Arg-1 expression (Figure 5H). We conclude that most, but not all, M1 markers are almost completely inhibited by curcumin treatment in stimulated microglial cells, whereas M2 markers are partially upregulated.

### Administration of Curcumin Significantly Reduces Pro-Inflammatory Cytokines in LPS + IFN-γ-Stimulated BV2 Cells

**Figure 6:**
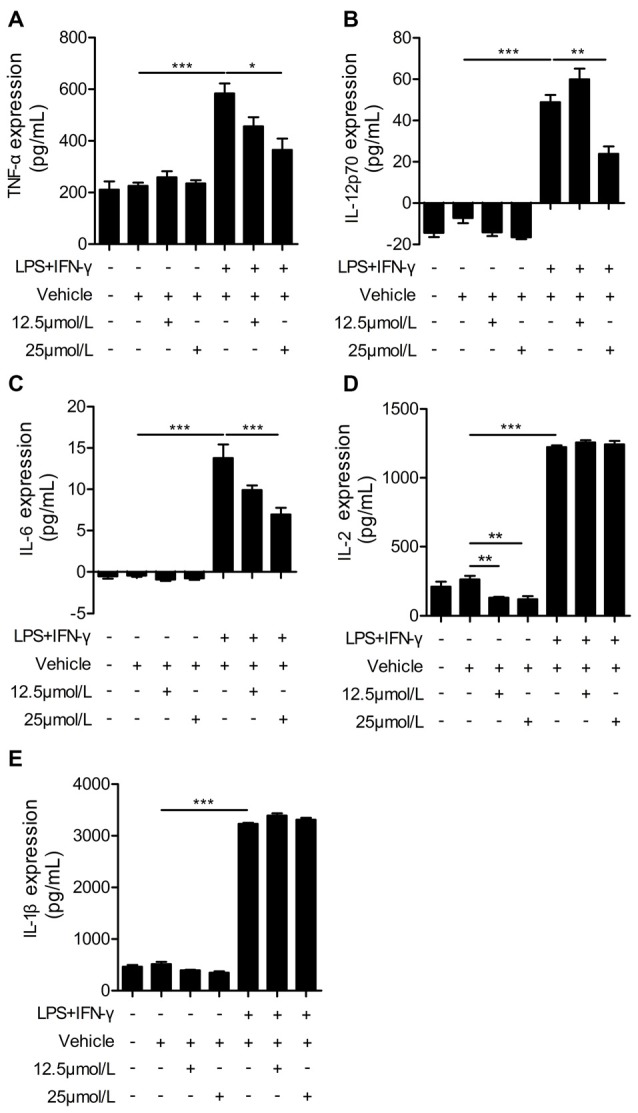
Curcumin suppresses inflammatory cytokines in LPS and IFN-γ-treated BV2 microglia. BV2 microglia were stimulated with LPS (100 ng/mL) and IFN-γ (20 ng/mL), and then treated with vehicle or 12.5 and 25 μmmol/L curcumin. The concentrations of inflammatory cytokines (TNF-α **(A)**, IL-12p70 **(B)**, IL-6 **(C)**, IL-2 **(D)**, and IL-1β **(E)**) were measured by ELISA. Data are means ± SEM. Samples were collected from six independent experiments, each performed in duplicate. *p < 0.05, **p < 0.01, ***p < 0.001, one-way ANOVA followed by Bonferroni *post hoc* test.

Next, we performed ELISAs to measure the release of pro-inflammatory cytokines in LPS + IFN-γ-stimulated BV2 cells. LPS and IFN-γ dramatically increased the production of pro-inflammatory cytokines from BV2 cells, including TNF-α, IL-12p70, IL-6, IL-2 and IL-1β (Figure 6). Curcumin treatment significantly reduced the production of pro-inflammatory cytokines including TNF-α, IL-12p70 and IL-6. However, the secretion of IL-1β and IL-2 was not dramatically attenuated with curcumin administration at 12.5 or 25 μmmol/L. Taken together, our *in vitro* data demonstrate that curcumin promotes M2 polarization and inhibits M1 polarization of microglia, and inhibits production of pro-inflammatory cytokines from M1-stimulated microglia. Collectively, these findings suggest a titration of polarization status by curcumin, both *in vivo* and *in vitro*.

## Discussion

Curcumin is a natural compound deemed as safe for ingestion by animals and humans by the Food and Drug Administration (FDA). The therapeutic potential of curcumin has been reported in many diseases, including cancer and neurodegenerative disorders (Pluta et al., 2015; Panda et al., 2017). A growing number of reports suggest that curcumin can protect against stroke-induced brain damage (Thiyagarajan and Sharma, 2004; Lin et al., 2016; Miao et al., 2016; Altinay et al., 2017). In the present study, we reported that curcumin treatment not only ameliorated infarct volumes, but also improved multiple aspects of neurological function at early timepoints after ischemic stroke.

Our previous study revealed an elevation of both M1 and M2 markers in microglia/macrophages after ischemic stroke, with an early M2-dominant phenotype and a later transition into M1-dominant responses (Hu et al., 2012). Different microglial phenotypes are well known to exert distinct effects on stroke pathology and brain repair (Hu et al., 2012, 2015). M1 microglia are known to release inflammatory cytokines (TNF-α, IL-1β, IL-6, iNOS and IL-12), which accelerate cell death and aggravate local inflammation (Girard et al., 2013). In contrast, M2 polarization upregulates anti-inflammatory or reparative factors and protects the ischemic brain. The lack of necessary endogenous signals for M2 induction is known to worsen outcomes after cerebral ischemia (Lee et al., 2016; Liu X. et al., 2016; Yang et al., 2017). For example, IL-4 deficient mice display less long-term functional recovery after ischemic stroke (Liu X. et al., 2016). Thus, shifting microglia from the M1 phenotype toward M2 may be an effective therapeutic strategy for ischemic stroke. Notably, we discovered that curcumin can shift microglia/macrophage polarization toward the neuroprotective and tissue-reparative M2 phenotype in the ischemic brain. *In vitro* studies in a microglial cell line confirm a direct effect on microglial polarization. Similarly, curcumin treatment inhibited cerebral inflammation in the ischemic brain *in vivo*. In agreement with our results, several recent studies demonstrated that curcumin induced macrophage polarization toward M2, and this was accompanied by an inhibition of local inflammatory responses (Gao et al., 2015; Karuppagounder et al., 2016).

Although the mechanisms whereby curcumin promotes microglial M2 polarization are not yet clear, several signaling pathways critical for microglial phenotype regulation may be activated by curcumin. For example, curcumin inhibited the phosphorylation and activation of STAT1 and STAT3 (Qin et al., 2012)—known to be important for microglial M1 polarization—in gangliosides, LPS, or IFN-γ activated microglia (Kim et al., 2003). In addition, miRNA 155 enhances M1 polarization and suppresses the expression of M2 signature genes (Cai et al., 2012; Moore et al., 2013) and is downregulated by curcumin treatment in LPS-treated macrophages and LPS-treated mice (Ma et al., 2017). Further studies are warranted to confirm the involvement of these mechanisms in curcumin-afforded neuroprotection and microglial phenotypic regulations in stroke models.

Similar to other polyphenols, curcumin is known to possess pleiotropic activities (Gupta et al., 2012). Previous studies performed in stroke models have shown that curcumin exerts diverse neuroprotective functions, including antioxidative effects (Thiyagarajan and Sharma, 2004; Yang et al., 2009; Wu et al., 2013), regulation of neuronal (and other cell) apoptosis (Zhao et al., 2008, 2010; Altinay et al., 2017), promotion of mitochondrial biogenesis (Liu et al., 2014), and enhancement of neurogenesis (Liu S. et al., 2016). The present study demonstrates that curcumin reduces ischemic stroke-induced brain damage and improves several functional outcomes. Our *in* *vivo* and *in vitro* studies suggest that curcumin has a profound regulatory effect on microglial responses, promoting M2 microglial polarization and inhibiting microglia-mediated pro-inflammatory responses. Thus, curcumin and perhaps other polyphenols may be “physiology tuners” that, among other beneficial functions, balance the equilibrium between M1 and M2 polarization states to elicit a wide range of protective effects. This multi-targeting characteristic of curcumin may underlie its efficacy in models of ischemic brain injury and justifies further investigation. Finally, the advent of new delivery systems might help increase the bioavailability and pharmacokinetic activity of curcumin (Panda et al., 2017), which would facilitate the translation of this compound to the clinic.

## Author Contributions

ZL and SH are responsible for the animal experiments. YR is responsible for the data acquisition and RT-PCR experiments. SW and XL performed the cell culture, ELISA and RT-PCR experiments. WZ performed the immunohistochemical staining experiments. XH, RKL, XG, ZJ, XJ and HD are responsible for the conception or design of the work. All authors drafted the work or revisited it critically and approved the final version of the manuscript.

## Conflict of Interest Statement

The authors declare that the research was conducted in the absence of any commercial or financial relationships that could be construed as a potential conflict of interest.
